# Using structure prediction of negative sense RNA virus nucleoproteins to assess evolutionary relationships

**DOI:** 10.1101/2024.02.16.580771

**Published:** 2024-02-17

**Authors:** Kimberly R. Sabsay, Aartjan J.W. te Velthuis

**Affiliations:** 1 Lewis Thomas Laboratory, Department of Molecular Biology, Princeton University, Princeton, NJ 08544, United States.; 2 Sigler Institute, Princeton University, Princeton, NJ 08544, United States.

**Keywords:** negative-sense RNA virus, NSV, nucleoprotein, NP, RNA polymerase, RdRp, phylogenomics, evolution, segmentation, genome, AlphaFold 2.0

## Abstract

Negative sense RNA viruses (NSV) include some of the most detrimental human pathogens, including the influenza, Ebola and measles viruses. NSV genomes consist of one or multiple single-stranded RNA molecules that are encapsidated into one or more ribonucleoprotein (RNP) complexes. Current evolutionary relationships within the NSV phylum are based on alignment of conserved RNA-dependent RNA polymerase (RdRp) domain amino acid sequences. However, the RdRp-based phylogeny does not address whether other core proteins in the NSV genome evolved along the same trajectory. Moreover, the current classification of NSVs does not consistently match the segmented and non-segmented nature of negative-sense virus genomes. Viruses belonging to e.g. the *Serpentovirales* have a segmented genome but are classified among the non-segmented negative-sense RNA viruses. We hypothesized that RNA genome segmentation is not coupled to the RdRp domain, but rather to the nucleocapsid protein (NP) that forms RNP complexes with the viral RNA. Because NP sequences are too short to infer robust phylogenetic relationships, we here used experimentally-obtained and AlphaFold 2.0-predicted NP structures to probe whether evolutionary relationships can be estimated using NSV NP sequences and potentially improve our understanding of the relationships between NSV subphyla and the NSV genome organization. Following flexible structure alignments of modeled structures, we find that the structural homology of the NSV NPs reveals phylogenetic clusters that are consistent with the currently accepted NSV taxonomy based on RdRp sequences with one key difference: the NPs of the segmented *Serpentovirales* cluster with the other segmented NSV. In addition, we were able to assign viruses for which RdRp sequences are currently missing to phylogenetic clusters. Overall, our results suggest that the NSV RdRp and NP genes largely evolved along similar trajectories, that NP-based clustering is better correlated with the NSV genome structure organization, and that even short pieces of genetic, protein-coding information can be used to infer evolutionary relationships, potentially making metagenomic analyses more valuable.

## Introduction

Negative sense RNA viruses (NSVs) are important human pathogens, and include the influenza A virus (IAV), Rift Valley fever virus (RVFV), Lassa virus (LASV), measles virus (MeV), rabies virus (RABV), and Ebola virus (EBOV). The genomes of NSVs consist of single-stranded, negative sense RNA that is copied in the context of a ribonucleoprotein (RNP) complex. Each RNP is composed of a viral RNA (vRNA) template, an RNA polymerase, and numerous nucleoproteins or nucleocapsids (NPs) (1, 2). The self-oligomerizing nucleoprotein (NP) molecules make up the majority of each RNP. These NPs protect the genome from degradation, act as scaffold for RNA structure formation, and assist in viral replication by acting as processivity factor (3, 4). Despite these conserved features, both the genome organization, and replication and transcription mechanisms vary widely among NSVs (5). Understanding the properties and evolutionary history of NPs may shed light on how different NP support NSV genome structures and RNA polymerase processivity.

**Figure 1:**
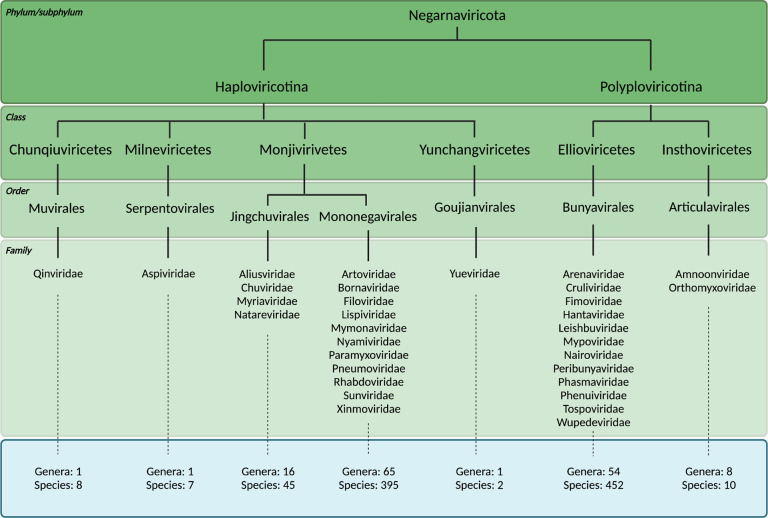
Classified viral species within *Negarnaviricota*. Taxonomic breakdown of the *Negarnaviricota* phylum of the *Riboviria* realm according to the ICTV and NCBI databases. The phylum consists of all NSVs and is divided into the subphyla *Haploviricotina* and *Polyploviricotina* based on the presence or absence of mRNA capping activity in the RNA polymerase and the organization (non-segmented or segmented) of the viral genome. Note that the *Serpentovirales* have a segmented genome, but an RNA polymerase with mRNA capping activity.

The International Committee on Taxonomy of Viruses (ICTV) established the phylum *Negarnaviricota* for negative sense RNA viruses (NSVs) in 2019 (Figure 1). Every virus within *Negarnaviricota* shares a common three-gene core: the RNA polymerase gene(s), membrane glycoprotein genes, and an NP gene (6). The RNA-dependent RNA polymerase (RdRp) domain of the RNA polymerase gene product is approximately 300 amino acids long and conserved amongst all RNA viruses (kingdom *Orthonavirae*), whereas the rest of the RNA polymerase is not. These characteristics have made the RdRp domain the focal point for evolutionary analyses (7, 8). The RdRp domain phylogenies have mapped out five major branches of *Orthonavirae* and elucidated ancestral trends amongst them. While traditional phylogenomics utilizes metagenomics data, sequence alignments, and comparison metrics, the ever-expanding wealth of structural information provides an avenue for the refinement of important branching points (9–17). In particular, structural comparisons of RdRp domains have illustrated surprising similarity amongst *Orthomyxoviruses* (with negative-sense ssRNA), flaviviruses (with positive-sense ssRNA) and *Cystoviruses* (with dsRNA), suggesting NSVs originated from dsRNA viruses which in turn evolved from positive-sense ssRNA viruses (14). NSVs subsequently evolved into subphyla (Figure 1), although the details of this split are unknown.

NSVs have historically been connected evolutionarily through the conserved RdRp domain (18). Based on the RdRp domain sequence, the NSV phylum is subdivided into *Haploviricotina* and *Polyploviricotina*. This division separates the NSVs that have a non-segmented genome and an RNA polymerase that possess mRNA capping activity from those that have a segmented genome and an RNA polymerase that performs cap-snatching to cap viral mRNAs, respectively (8). Genome segmentation allows for reassortment of gene segments, which can contribute to global pandemics and the spread of immune or antiviral resistance mutations (19, 20). Segmentation also makes the differential expression of viral genes easier to regulate (21, 22). On the other hand, segmentation complicates virus genome packaging. A virion of a segmented virus is not viable unless it contains the entire set of viral genome segments. The coordination and packaging of all segments is thus a crucial step in segmented NSV infections. In influenza A viruses, the majority of virion particles contain one copy of each of the eight genome segments (23).

**Figure 2:**
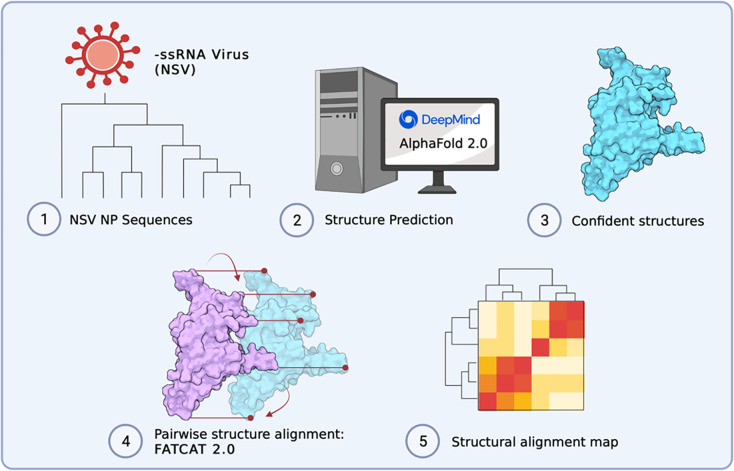
Computational pipeline to build structural alignment map. (**1–2**) Data was acquired based on sequence availability. AlphaFold 2.0 was used to predict structures. (**3–4**) AlphaPickle was used to visualize confident metrics, following which structures were selected based on confidence score cut-offs and individual pairwise flexible alignments were computed using the FATCAT 2.0 package compiled on a high performance cluster (HPC). (**5**) Data processing was performed to parse structural alignment data into a concise heatmap for comparison with MSA percent identity matrices.

Previous work has used analysis of RdRp domain sequences to understand the evolution of genome structure and segmentation within *Negarnaviricota* or the relations among NSV orders (6, 24). The important role of NPs in vRNA protection, replication, and packaging, as well as in RNP morphology, suggests that the evolutionary history of NPs may reveal additional insights into the evolution of NSV genome segmentation. However, NP sequences are too short for robust phylogenetic analyses. We here combined experimentally obtained structural data and the deep learning tool AlphaFold 2.0 (AlphaFold2) (Figure 2) to explore if the evolutionary relationships among NSVs can be reconstructed from NP sequences and if this relationship provides additional insight into the divergence of the NSV subphyla and the emergence NSV genome segmentation. In addition, this approach may be useful for metagenomics and virus discovery studies where RdRp sequences may not be complete or missing. We find that the structural homology of NPs within NSVs provides more phylogenetic information than NP sequences alone. In addition, we observe that the NP structural clustering largely matches the clustering and known inferred phylogeny of the RdRp domain, with the exception of the segmented *Serpentovirales*, suggesting that the two NSV core genes largely co-evolved. The clustering of the *Serpentovirales* with the other segmented NSVs, instead of the non-segmented NSVs, suggest that the NP structure-based clustering is better correlated with the division between the segmented and non-segmented NSV genome organization than the RdRp-based phylogeny.

## Results

### Experimental NP structure dataset

**Figure 3:**
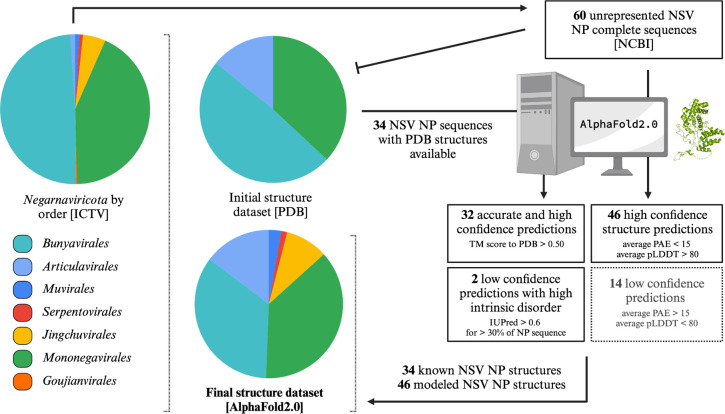
Flowchart for the creation of a representative structural dataset of Negarnaviricota NPs. The composition breakdown of *Negarnaviricota* by phylogenetic order (*left*) was determined by the number of species in each order in the ICTV/NCBI database at the time of the manuscript preparation. The percentages of each order are as follows: 49.2% *Bunyavirales*, 1.0% *Articulavirales*, 0.9% *Muvirales*, 0.8% *Serpentovirales*, 4.9% *Jinchuvirales*, 43.0% *Mononegavirales*, and 0.2% *Goujianvirales*. At the time of the manuscript preparation, there were 34 NSV NP structures available in the PDB database with representation from only the *Bunyavirales*, *Articulavirales,* and *Mononegavirales*. The final NSV NP dataset included a total of 80 structures generated with AlphaFold 2.0.

At the time of submission of this manuscript, there were 457 viral species in the *Haploviricotina* and 462 species in the *Polyploviricotina* assignments of the ICTV database. The taxonomical breakdown is summarized in Figure 1. Structural data for NPs was found for 34 of these NSVs in the PDB database. Within this initial dataset, 21 NP structures were from *Polyploviricotina* and 13 from *Haploviricotina*. Given the relatively even ratio of officially classified viral species in each subphyla in the ICTV, this initial NP structure dataset appears to be biased towards the human-disease causing, segmented NSVs (Figure 3).

### NP structure prediction using AlphaFold2

**Figure 4:**
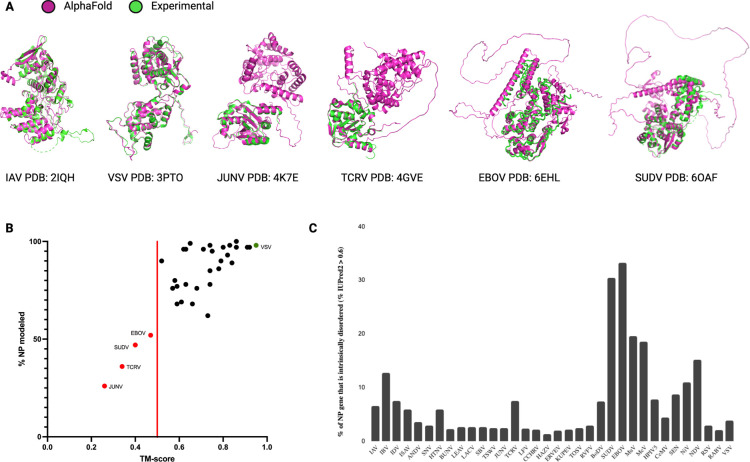
AlphaFold2 structure prediction performance on solved NSV nucleoproteins. Overall prediction performance of AlphaFold 2.0 on NSV NPs was assessed using an initial dataset of 34 NSVs with experimentally solved structures deposited in PDB. (**A**) Structural alignments of AlphaFold2 predictions (magenta) and experimental structures (green) are displayed for the most accurate prediction (VSV and IAV, left), a partially resolved experimental structure with the lowest % of NP modeled (JUNV and TCRV, middle), and a highly intrinsically disordered structure (SUDV and EBOV, right). (**B**) The predicted AlphaFold 2.0 structures were compared to the experimental NP structures using rigid jFATCAT structural alignment and plotted according to the alignment score (TM-score). Four structures fell below a TM-score of 0.50 (red line) and are depicted as red points and labeled. The top scoring AlphaFold prediction with an almost perfect alignment (VSV NP) is denoted by the green data point. (**C**) Intrinsic protein disorder predictions for the initial dataset using IUPred2 show a high percentage of disorder within the EBOV and SUDV NPs.

Phylogenetic analyses can be performed on many types of information, including multiple-sequence alignments and structural alignments. We wondered if the current NP structure dataset could be expanded using structural prediction methods like AlphaFold2. To explore this, we first determined if AlphaFold2 could accurately predict the structures of the known NPs based on the primary sequences of the 34 NP structures in the PDB. To minimize the impact of the known NP structure on the prediction, the AlphaFold2 settings were restricted to structure templates released prior to the release dates of the NP structures in the PDB. As shown in Fig. 4A, the overall performance of AlphaFold2 was robust, with 27 out of 34 predictions having high overall model confidence (average pLDDT scores above 70) (Supplementary raw data file 1). Aligning the predicted structures to the corresponding experimental structures produces TM-scores above the 0.50 threshold for 30 out of 34 structures, suggesting that the predictions are in fact of the same general topology as the experimental structures (25).

Next, we aligned the AlphaFold2 structures with the corresponding experimental structures using jFATCAT. Overall, we find good agreement between the experimental and computed structures, with the VSV NP showing the best agreement with a TM-score of 0.95 (Figure 4A). However, for some NPs we observed deviations in regions of computed intrinsic disorder, which many experimental structure-solution methods were unable to resolve (Figure 4A). Only four structures seemed to have a low resemblance to the experimental structure, with TM-scores less than 0.50 (Figure 4B). Further analysis showed that the TM-score of the AlphaFold2 structure correlated with the percentage of modeled NP residues and/or the percentage of sequence coverage of the experimental structure (Figure 4B). Thus the experimental NP structures with limited sequence coverage or missing residues (i.e., the Junin arenavirus (JUNV), Tacaribe virus (TCRV), Sudan ebolavirus (SUDV), and Zaire ebolavirus (EBOV) NPs) align poorly to the AlphaFold2 predictions of the complete NP sequences.

Investigating the four, relatively poorly aligned NP models in more detail, we find that both the experimental JUNV NP structure (PDB: 4K7E) and the TCRV NP structure (PDB: 4GVE) contain only the C-terminal domains (Figure 4A), while the AlphaFold2 models contain both C-terminal and N-terminal domains. Superposing the partial experimental NP structures with the AlphaFold2 models shows good alignment between the C-terminal domains (Figure 4A). The remaining two outliers, EBOV NP (PDB: 6EHL) and SUDV NP (PDB: 6OAf), have experimental structures of which only the N-terminal core domain is resolved. The unresolved C-terminal domains of both EBOV and SUDV NPs are predicted to be intrinsically disordered domains. The AlphaFold2-predicted structures align with the N-terminal core domains that are resolved in the experimental structures, while the parts of the AlphaFold2-predicted structure that do not align contain large unstructured loops that have low prediction confidence (Figure 4A). The AlphaFold2 predictions follow the IUPred2 protein disorder prediction for the sequences in their entirety (26) (Figure 4C). The unstructured regions and low-confidence loops in the AlphaFold2 predictions correlate with the regions of high disorder prediction (regions longer than a few amino acids with disorder prediction scores above 0.50). Analysis of the predicted disordered regions in the 34 NP models shows that large regions of intrinsic disorder (>20%) are uniquely present in the *Filoviridae* NPs (Figure 4C). These disordered regions are likely inherent properties of individual NPs and not an erroneous artifact of the AlphaFold2 analysis. We thus consider the AlphaFold2 predictions sufficiently robust for the majority of NP structures to expand the experimental NP structure dataset.

### Expanding the NP structure dataset using AlphaFold2

We next used AlphaFold2 to extend the experimental NP structure dataset to obtain better structural coverage of the *Negarnaviricota* NPs. To this end, additional viral species with known and complete NP sequences were chosen from unrepresented NSV families and the NP structures of these viruses predicted using AlphaFold2. The structure predictions that passed the confidence requirements (average PAE scores <15 and average pLDDT scores >80) were included in the final NP structure dataset (Figure 3). This dataset contained a total of 80 NP structures with 50.6% from *Haploviricotina* and 49.4% from *Polyploviricotina* (Supplementary Table 1).

### Analysis of the NP and RdRp domain MSAs

**Figure 5:**
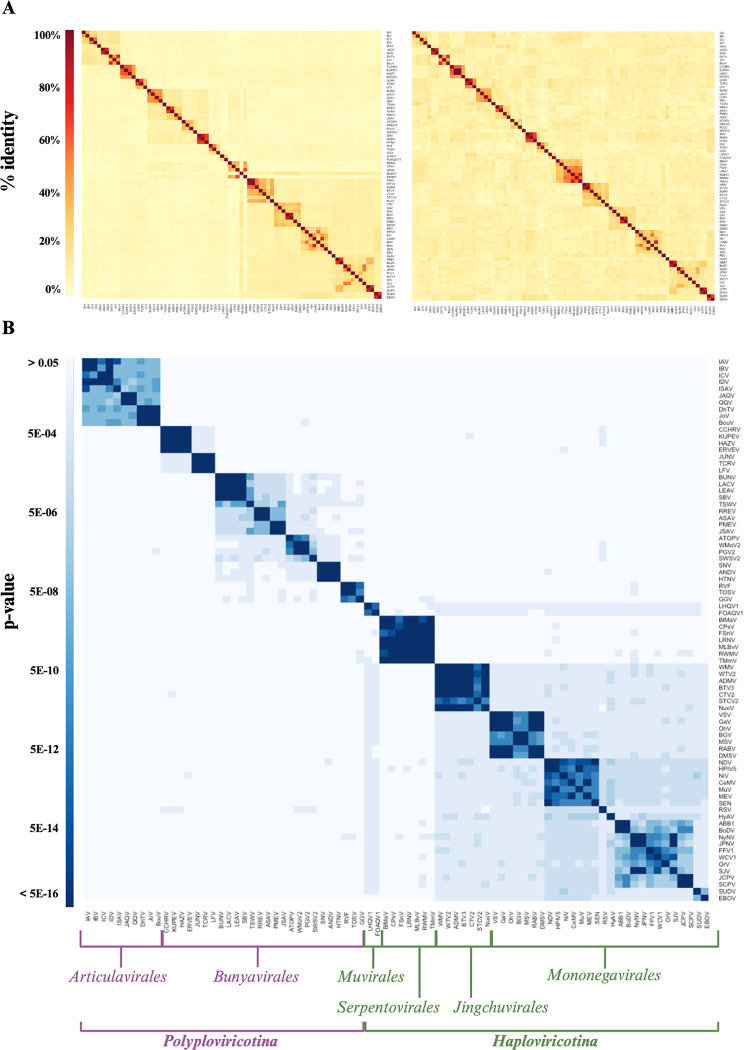
NSV NP structural alignment matrix matches RdRp MSA matrix. (**A**) RdRp (*left*) and NP (*right*) amino acid sequence MSAs illustrated as percent identity matrices. Note that the RdRp MSA did not include 2 of the 80 NSVs used in other matrices, as freesia sneak virus (FSnV) and tulip mild mottle virus (TMmV) RdRp sequences were available in sequence databases at the time of analysis. (**B**) NP structural alignment by FATCAT in an order consistent with the heatmaps in panel A, and illustrated as a p-value matrix.

To assess if the NP structures contained enough information to investigate the evolutionary history of NP, we first generated an MSA of the RdRp domain sequences to use as a benchmark. Only 78 of the 80 NSVs were included in this MSA as the freesia sneak virus (FSnV) and tulip mild mottle virus (TMmV) RdRp sequences were not available in sequence databases at the time of analysis. The MSA rows were arranged according to the current ICTV classification and the resulting MSA used to generate a percent identity matrix. This matrix is shown as a heatmap in Figure 5A, left. We next generated an MSA using the NP sequences and illustrated the NP percent identity matrix as a heatmap as well (Figure 5A, right).

The RdRp and NP heatmaps shown in Figure 5A are presented with an identical x- and y-axis order, with the exception of FSnV or TMmV, which are absent in the RdRp MSA heatmap. The clustering can thus be qualitatively compared across the two alignments. However, it is important to note that the color scales of the MSA and FATCAT heatmaps should not be directly compared as they derive from different alignment calculations. The predefined order of the heatmaps separates the segmented NSVs (first 36, starting left) from the non-segmented NSVs (last 44). The heatmap that is based on the RdRp MSA shows four dark clusters in the top left corner that are grouped into a single larger cluster. This larger group consists of 10 NSVs that all belong to the *Articulavirus* order. The four smaller clusters represent the *Influenza virus* (4), *Quaranjavirus* (2), *Isavirus* (1), and *Thogotovirus* (3) genera, in line with the current ICTV phylogeny. Comparing the RdRp and NP MSA heatmaps, we note that while the four virus family clusters are visible in the NP heatmap, the virus order clustering seen in the RdRp heatmap is lost in the NP heatmap.

In the RdRp heatmap, the next two darker clusters correspond to the four *nairoviruses* and three *arenaviruses*, which all belong to the *bunyavirus* order. The NP heatmap shows the same *nairovirus* and *arenavirus* clustering, but neither heatmap clusters the two families into the same order or even a larger cluster. The next clusters in the RdRp heatmap are made up of 16 additional *bunyaviruses* that fall into the five viral families *Peribunyaviridae* (4), *Tospoviridae* (1), *Fimoviridae* (4), *Phasmaviridae* (4), and *Hantaviridae* (3). The RdRp and NP heatmaps cluster these 16 viruses into the same five families as the current taxonomy, but only the RdRp heatmap clusters them into a single larger cluster. The three remaining bunyaviruses in our data set belong to the *Phenuiviruses* family. We find them clustered adjacent to the above larger cluster of five bunyavirus families. We do not observe a single cluster that perfectly encapsulates all members of the *Bunyavirus* order.

From GGV onwards, we find a small cluster of two *Muvirales* and a larger cluster of *Serpentoviruses* in the RdRp (5) and NP heatmaps (7). The RdRp heatmaps clusters 4 of the 5 *Serpentoviruses* with the larger cluster of six *Bunyaviruses* families (the *Nairovirus* and *Arenavirus* are not included in this cluster). The exception is *Mirafiori lettuce big vein virus* (MLBvV), which the RdRp heatmaps clusters with a large *Haploviricotina* (non-segmented NSVs) cluster. In the NP heatmap, this clustering is not observed. The NP heatmap does show clustering of FSnV or TMmV, for which RdRp domain sequences were not available, with the *Serpentoviruses*. It is important to note here that, at the time of analysis, the *Serpentoviruses* are taxonomically classified within *Haploviricotina* based on the mRNA capping ability of the RNA polymerase even though their genomes are segmented. The RdRp domain MSA suggests that in spite of the capping domain, their NTP incorporation function may be more closely related to the *Bunyavirales*.

The second part of the heatmaps consists of 35 non-segmented NSVs that together form the clusters: *Rhabdoviruses* (7), *Chuviruses* (7), *Paramyxoviruses* / *Pneumovirus* (8), Qinvirus / Lispivirus (3), *Nyamiviruses* (6), *Bornaviruses* (2), and *Filoviruses* (2). In the RdRp domain heatmap, these seven clusters are combined into one large cluster, but this clustering is not evident in the NP heatmap. Overall, this analysis suggests that the virus family clusters are resolved in both the RdRp and NP heatmap and that the family clustering is consistent with the current phylogeny of *Negarnaviricota* (27, 28). However, at a higher level, the RdRp domain heatmap is able to identify some orders that the NP heatmap can not. Thus the RdRp domain sequence appears to offer more phylogenetic information to derive long-distance ancestry than the NP sequence.

### Pair-wise analysis of NP structures is comparable to RdRp domain MSA

We hypothesized that the NP AlphaFold2 structure dataset contained more phylogenetic information than the NP sequences alone, because protein structures are generally more conserved than the primary sequence. To explore this, we had to align our AlphaFold2 NP structures. Aligning structural information requires a completely different approach and significantly more computational power than aligning primary amino acid sequence data. Foundational structural alignments utilize rigid body backbone algorithms. While this is a powerful approach for assessing the similarity of closely related structures that are solved in similar conditions, the natural motion and dynamics of protein structures are not accounted for in rigid body alignments (29, 30). As proteins can adopt multiple conformations according to their function or protein structures have different energy minima, it is critical to analyze structures in a way that tests whether two proteins identical, even if they have adopted different conformations (e.g. “open” or “closed”, or “active” or “inactive”). Various complex algorithms have been developed to attempt to incorporate dynamic motion into structural alignments. For our NP AlphaFold2 structure analyses, we used the Flexible structure AlignmenT by Chaining Aligned fragment pairs allowing Twists (FATCAT) algorithm (31). This method utilizes fragment pairs and iterative rotations/twists to converge on an alignment that would account for alternate conformations. As is the case with NSV NPs, the two-domain NP structures have been proposed to twist upon oligomerization and/or RNA-binding to form the helical chains seen in RNP complexes (32–35).

Pairwise alignments for all possible pairs of NPs within the dataset were individually computed. Each alignment pair resulted in an alignment p-value, which we interpret as the probability of observing a more identical alignment between the structure pairs. A structural alignment heatmap was generated from the alignment p-values and organized in the same predefined order as illustrated in the MSA heatmaps for direct comparison (Figure 5C). The diagonal represents self alignment, or a p-value of exactly zero. A computational pipeline integrates AlphaFold2, AlphaPickle, FATCAT 2.0, and original data processing and screening code, to allow for high-throughput expansion of the pairwise structural alignment map described above (Figure 2) (13, 31, 36).

The first main cluster of the NP structure alignment is in agreement with the RdRp MSA, containing the 10 *articulaviruses*/*orthomyxoviridae*. The order/family cluster can be divided into four sub-clusters that match the four genera in our dataset. The following two main structure alignment clusters are also consistent with the RdRp MSA and correspond to the *nairovirus* and *arenavirus* clusters. The remaining 19 bunyaviruses form one larger cluster that contains subclusters that match the six bunyavirus families. The NP structural alignment thus outperforms the NP MSA and generates a larger bunyavirus cluster that is not observed in the RdRp MSA.

The *Muvirales* follow the *Bunyavirales* in the NP structure alignment heatmap. Interestingly, the two included NPs appear to cluster with the large *Haploviricotina* cluster. The large cluster that follows the Muvirales in the NP structure alignment corresponds to the *Serpentoviruses*. In the RdRp MSA, MLBvV seemed to deviate from the other *Serpentoviruses* and cluster with the non-segmented viruses, whereas the other *Serpentoviruses* appeared to cluster with the *bunyaviruses*. In the NP structure alignment, MLBvV does not appear to be an outlier, but clusters tightly with the other *Serpentoviruses*, and remains separate from the non-segmented NSV cluster. The final main cluster within the NP structure alignment corresponds to the remaining non-segmented NSVs. This observation is fascinating as it contradicts the current classification of the *Serpentoviruses* as a member of *Haploviricotina* based on RdRp function and phylogenetic analyses and suggests for these viruses RdRp and NP functions diverged, which aligns with the capping capabilities of the RNA polymerase in combination with the segmented genome (37).

Overall, NPs encoded by viruses in the non-segmented NSV subphylum have a consistently high structural similarity with each other, while NPs encoded by viruses of the segmented NSV subphylum display greater structural variability and distinction between viral families, which is consistent with previous hypotheses (2). The fact that *Serpentoviruses* appear to encode NPs with structures that are structurally more consistent with NPs encoded by viruses from the segmented subphylum is a new insight and contradicts analyses performed using RdRp sequences. The currently accepted classification of NSVs fails to accurately differentiate NSV based on the structural organization of their genetic material. Our new analysis based on NP structural modeling reflects clustering consistent with the NSV genome organization.

## Discussion

Phylogenomic analyses of RNA virus sequences are typically performed by comparing RdRp domain amino acid sequences. A Clustal Omega-generated MSA of the NSV RdRp sequences shows a clustering that is consistent at the family level with the phylogenetic classification currently used by the ICTV (Figure 5A) (27, 28). However, the RdRp MSA does not include all possible NSV sequences available. For instance, FSnV or TMmV are missing because complete genome sequences, and in particular the RdRp domain sequences, are not available at this time. When an NP MSA was performed for the same viruses as the RdRp MSA, the percent identity matrix showed some clustering of closely related NSVs, consistent with clustering seen in the RdRp MSA. However, any resolution of the virus order and class was lost in the NP MSA (Figure 5A). This observation is in agreement with other studies showing that the RdRp domain sequences provide the most evolutionary information for viral genome analyses. While sequence alignment helps us estimate the evolutionary relationships within NSVs, no previous work has been done to explore the potential of utilizing structural data to assess NSV phylogenetic patterns. It is well acknowledged that proteins are more conserved at the structural level than at the sequence level (11, 12). As structural data is becoming more readily available, the wealth of information it provides should be incorporated into our understanding of the functional conservation of viral proteins throughout time. Indeed, recent studies have shown that structure-based phylogenetic analyses can outperform sequence-based phylogenetic analyses (9–17).

By converting the NSV NP sequence dataset into an AlphaFold2-derived structural dataset using flexible alignment, similar clustering patterns to the RdRp domain MSA emerged (Figure 5B). However, we did observe key differences. Of particular interest is the phylogenetic relationship linking the segmented *Serpentovirales* to the other segmented NSVs instead of the non-segmented NSVs. This observation suggests that the current classification of the *Serpentovirales* may need to be critically examined. Electron microscopy analyses have shown that *serpentovirus* RNPs are flexbile, having internally coiled loop-like structures, similar to the *bunyavirus* RNPs, and more linear collapsed duplex structures, similar to *articulavirus* RNPs (40). Morphologically, *serpentovirus* RNPs most closely resemble RNPs of viruses in the *tospoviridae* family (of the *bunyavirus* order), yet there is no evidence that serpentoviruses have enveloped virion particles and, as is the case for *tospoviruses*. Previous analysis of the overall architecture of NSV RNPs has shown a pattern in RNP flexibility that corresponds directly to genome organization (4). However, since there are only two micrographs available for *serpentovirales* RNPs, it is possible that this observation is an outlier rather than consistent across the seven species. Obtaining additional electron micrographs is need to support a phylogenetic classification based on genome morphology and/or structure.

The above observations make it tempting to speculate about the evolutionary origin of NSV genome segmentation. The viral families that have been previously hypothesized to be associated with the evolution of NSV genome segmentation include *serpentoviruses* (within the *Ophioviridae*) along with viral species that belong to the *Chuviridae* (6). While all currently identified species of *Serpentovirales* have segmented genomes, *Chuviriales* with non-segmented and segmented genomes have been identified. Our current dataset includes 7 non-segmented *Chuvirales* that all belong to the *Jingchuvirales* order. These viruses firmly cluster with the nonsegmented NSVs in line with their genome organization. To obtain a better sense where the segmented *Chuvirales* would cluster, and potentially their position relative to the origin of NSV genome segmentation, our NP structural analysis can be extended in the future. Further analysis of the *Serpentovirales* may also provide greater insight into our understanding of the evolution of segmented NSV genomes.

It is possible that segmented NSV genomes evolved multiple times. This hypothesis could explain the large differences seen between articulaviruses and bunyaviruses in terms of the number of genome segments and the gene organization in the respective viral genomes. Moreover, several viral species that belong to the *monogenavirales* have segmented genomes, including orchid fleck dichorhavirus, which is classified within the *rhabdoviridae* and possesses two genome segments (6). Phylogenetic analysis suggests that this virus evolved from a non-segmented plant virus within the *rhabdoviridae* (39).

Lastly, we need to consider the likelihood that a multitude of viral species remains to be identified, and that the “origin species” of NSV genome segmentation has not been discovered yet. It is also possible that this species has been identified, but that it has so far been excluded from large scale evolutionary analyses because RdRp sequences were incomplete or missing. In particular for the latter aspect, the findings presented here suggest a useful role for including structural information based on sequences of non-RdRp proteins, such as NP in phylogenomic analyses. We therefore hope that our findings will help inspire new research and ultimately the identification of a possible origin of NSV genome segmentation.

## Materials and Methods

### Data Acquisition

Sequences and currently accepted taxonomic classifications were acquired from ICTV and NCBI sequence databases. Accession numbers are available in Supplementary Table 1.

### Clustal Omega MSA

The EMBL-EBI Clustal Omega Multiple Sequence Alignment web server was used to perform MSA analysis for both the RdRp and the NP alignments (41). Protein sequences were consolidated into a single FASTA file and run with ClustalW output parameters and restricted to the predefined order of input. The percent identity matrix was used to make the MSA heatmaps in R.

### AlphaFold 2.0 structure prediction

The newest AlphaFold algorithm (version 2.0) was used on the Della HPC at Princeton University to predict the structures of all NPs within the dataset (13). The general performance of AlphaFold with viral NPs was assessed using the 35 known NP structures deposited in the PDB. The amino acid sequences of the viral NPs were used as the inputs and a maximum PDB template date was defined to be before the release date of each solved structure to ensure AlphaFold2 could not “cheat” during prediction.

The predicted NP models were then assessed for confidence (with both pLDDT and PAE metrics) using AlphaPickle (36). The predicted structures were then compared to the experimentally solved structures deposited in the PDB and all predicted NP structures were determined to be statistically accurate. The following structural analyses with the expanded dataset (including 80 NSVs) used all AlphaFold predicted NP structures to reduce structural solution method bias.

### Intrinsic protein structure disorder prediction

The IUPred package was downloaded from https://iupred.elte.hu/ to allow for faster data acquisition (26). Single sequences can be analyzed through the freely accessible web-server.

### FATCAT 2.0 Structure Alignment

The source code for Flexible structure AlignmenT by Chaining Aligned fragment pairs allowing Twists (FATCAT) was retrieved from the GodzikLab github repository (31). The software was compiled and built on the Della HPC at Princeton University. Original data organization code produced an input list of NP pairs in the predefined order. The FATCAT software then used the AlphaFold predicted NP structures to run flexible structure alignment for the 6,400 defined pairs. The results were quantified as a p-value, or the probability of observing a more identical alignment between the structure pairs. The alignment data was illustrated as a heatmap to facilitate clustering comparison to the MSA percent identity heatmaps.

### Alignment analysis in R

Original code to process and visually analyze the resulting alignment data was written in R. Heatmaps were created using R package gplots.

## Supplementary Material

Supplement 1 Supplementary Table 1: Full NSV Dataset

Supplement 2 Supplementary File 1: Initial Dataset NP PDBs (.pdb files)

Supplement 3 Supplementary File 2: Full NSV Dataset NP AlphaFold PDBs (.pdb files)

## Data Availability

Predicted AlphaFold structure files are available in pdb format in Supplementary Files 1 and 2. Dataset acquisition codes can be found in Supplementary Table 1.
